# iForensic, multicentric validation of digital whole slide images (WSI) in forensic histopathology setting according to the College of American Pathologists guidelines

**DOI:** 10.1007/s00414-025-03421-5

**Published:** 2025-01-21

**Authors:** Nicola Pigaiani, Antonio Oliva, Vito Cirielli, Simone Grassi, Vincenzo Arena, Luca-Maria Solari, Naomi Tatriele, Dario Raniero, Matteo Brunelli, Stefano Gobbo, Aldo Scarpa, Liron Pantanowitz, Pamela Rodegher, Federica Bortolotti, Francesco Ausania

**Affiliations:** 1 Unit of Forensic Medicine, Department of Diagnostics and Public Health, University of Verona, Verona, Italy; 2 Section of Legal Medicine, Department of Health Surveillance and Bioethics, Catholic University of Sacred Heart, Rome, Italy; 3 Unit of Forensic Medicine, Department of Prevention ULSS 8 Berica, Vicenza, Italy; 4 Section of Forensic Medical Sciences, Department of Health Sciences, University of Florence, University of Florence, Florence, Italy; 5 Institute of Anatomical Pathology, Department of Woman and Child Health and Public Health, Catholic University of Sacred Heart, Rome, Italy; 6 Unit of Pathology, Department of Diagnostics and Public Health, University of Verona, Verona, Italy; 7 Department of Pathology, University of Pittsburgh Medical Center, Pittsburgh, PA USA

**Keywords:** Digital forensic histopathology, Digital glass, Digital whole slide images, Forensic pathology, Method validation

## Abstract

Pathology has benefited from the rapid progress of image-digitizing technology during the last decade. However, the application of digital whole slide images (WSI) in forensic pathology still needs to be improved. WSI validation is crucial to ensure diagnostic performance, at least equivalent to glass slides and light microscopy. The College of American Pathologists Pathology and Laboratory Quality Center recently updated internal digital pathology system validation recommendations. Following these guidelines, this pilot study aimed to validate the performance of a digital approach for forensic histopathological diagnosis. Six independent skilled forensic pathologists from different forensic medicine institutes evaluated 100 glass slides of forensic interest (80 stained with standard hematoxylin and eosin, 20 with special staining), including different organs and tissues, with light microscopy (Olympus BX51, Tokyo, Japan). Glass slides were scanned using the Aperio GT 450 DX Digital Slides Scanner (Leica Biosystems, Nussloch, Germany). After two wash-out weeks, forensic pathologists evaluated WSIs in front of a widescreen using computer devices with dedicated software (O3 viewer, O3 Enterprise, Zucchetti, Trieste, Italy). Side-by-side comparisons between diagnoses performed on tissue glass slides versus WSIs were above the threshold stated in the validation guidelines (mean concordance of 97.8%). CSUQ Version 3 questionnaire showed high satisfaction for all pathologists (mean result: 6.6/7). Our institutional digital forensic pathology system has been validated for practical casework application. This approach opens new scenarios in practical forensic casework investigations, such as sharing live histological ex-glass slides online, as well as educational and research perspectives, with improving impacts on the whole daily workflow.

## Introduction

The crucial role of systematic histological examination in the medico-legal field, especially in forensic autopsies, has been gradually established over the last two decades [1]. Histology and histopathology can assist forensic scientists in many forensic contexts. Conventional histological techniques, such as hematoxylin–eosin (H&E) staining and special stains like histochemical stains and immunohistochemistry (IHC), play a pivotal role in forensic investigations. These techniques are applied to a wide range of sample types, including human hard tissues like bones, human soft tissues, and diatoms, being able to provide insights into various aspects, such as the age and vitality of lesions (whether they are acute, subacute, or chronic, as well as antemortem, perimortem, or post-mortem) and the determination of the cause of death, distinguishing between natural and non-natural causes [2].

Since the late 1990s, digital whole slide imaging (WSI) has become increasingly integrated into pathology practice, introducing the concept of using digital scans of histological glass slides in diagnostic processes. Initially, the first whole slide scanners (WSS) were rudimentary, taking significant time to acquire images and producing large files (approximately 7 Gigabytes of data for each focal plane). Nevertheless, advancements in automation, scanner technology, file compression systems, high-quality lenses and objectives, enhanced camera sensors, and expanded digital storage capacities have greatly improved the usability and accessibility of WSIs, making them more appealing in the market [3, 4]. The advent of digitized images, technical advances in scanning speed, and decreased costs have driven traditional pathology into the “digital pathology” era. Nowadays, pathology diagnosis on computer monitor screens using WSIs has been approved by the Food and Drug Administrations of the United States of America, the European Union, and Japan [5–7].

Despite the exponential growth of digital pathology literature, although histopathology is almost always performed within the forensic pathology field, WSIs in the forensic context have yet to gain significant traction. Given the extensive potential of this innovative technique for service, education, and research applications, it is noteworthy that, to the best of our knowledge, only two forensic pathology publications utilizing WSIs have been published to date [8, 9].

Given the importance of the validation step for routinely applying this technology to practical casework, the expert non-vendor panel convened by the College of American Pathologists Pathology and Laboratory Quality Center published in 2013 and recently updated practice guidelines for internal digital pathology system validation, recommended the evaluation of intra-observer diagnostic concordance between glass slides and WSIs in at least 60 routine cases, viewed firstly using a light microscope and after two wash-out weeks, using a digital viewer; the guidelines stated that diagnostic concordance between digital and glass slides for the same observer should be above 95% [10, 11].

Following these guidelines, this study aimed to validate the multicenter performance of a digital approach for forensic histopathological diagnosis, ensuring accuracy at least equivalent to that obtained by viewing glass slides through light microscopy.

## Material and methods

Six independent skilled forensic pathologists from different forensic medicine institutes evaluated 100 histopathological glass slides with light microscopy (Olympus BX51, Tokyo, Japan). Forensic pathologists were selected based on their experience: all physicians should have performed more than 500 complete autopsies, including histopathology analyses, before the study started. Glass slides were anonymized by applying a progressive alphanumeric code.

All glass slides analyzed in the first phase were scanned using the Aperio GT 450 DX Digital Slides Scanner (Leica Biosystems, Nussloch, Germany). Digital files were named using the same progressive alphanumeric code assigned to the original glass slides. All files were uploaded to a local server, and each pathologist was provided with confidential access to the server to recover images.

**Fig. 1:**
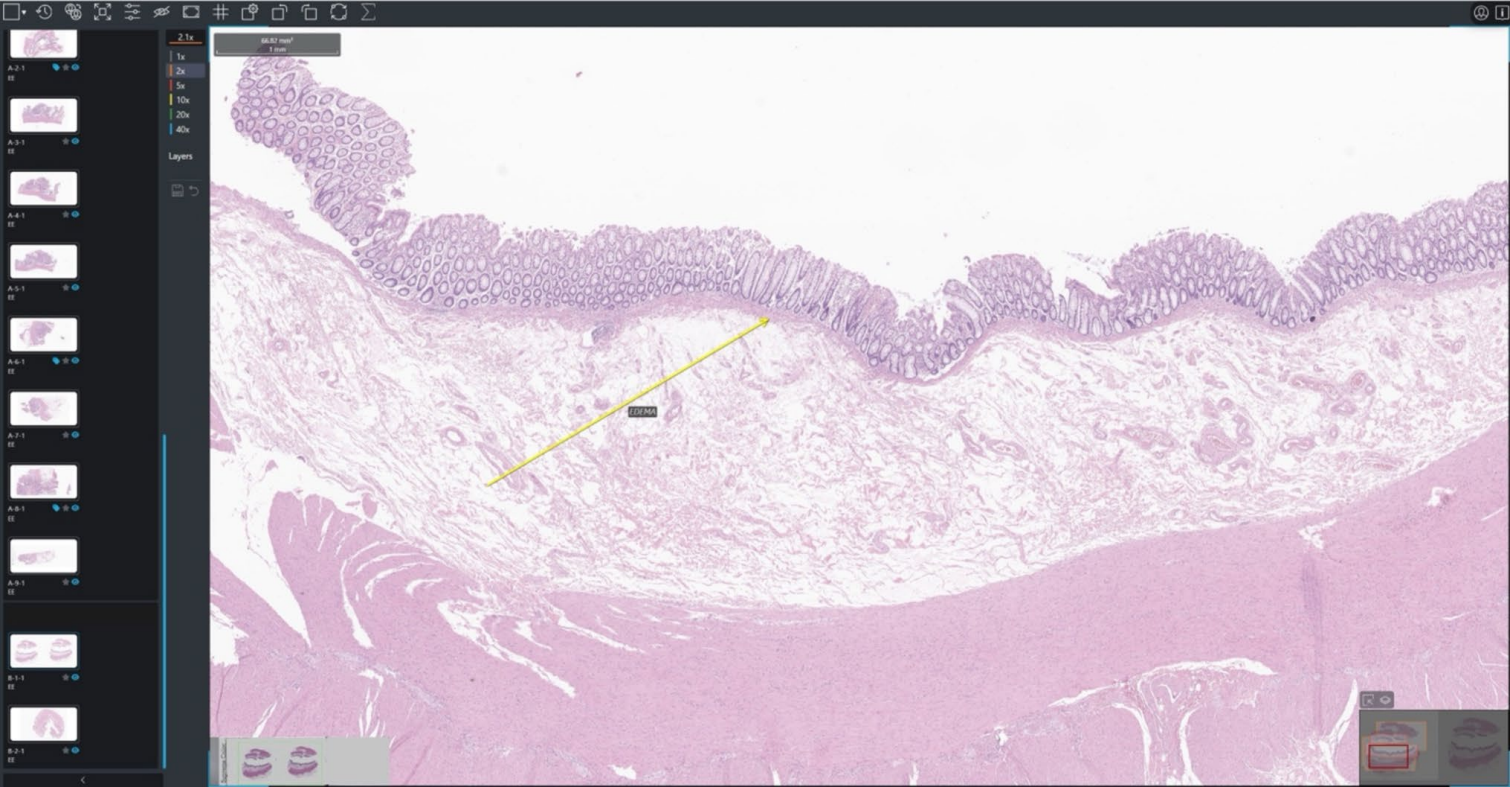
Digital environment for WSIs analysis. The digital glass holder is shown on the left side. The digital slide and the WSI portion viewed are shown at the bottom right

After two wash-out weeks, forensic pathologists evaluated WSIs in front of a widescreen using a specific digital viewer—O3 viewer (O3 Enterprise, Zucchetti, Trieste, Italy) shown in Fig. 1. Quality indicators related to images, usability, and workflow were recorded based on pathologists’ subjective perceptions (high versus poor).

A further independent pathologist check compared each pathologist’s diagnosis on glass and WSI to evaluate intra-operator concordance.

At the end of the procedure, the CSUQ Version 3 questionnaire was administered to study perceived usability. The original CSUQ Version 3 questionnaire established values between 1 and 7 or non-applicable (NA), with lower scores indicating higher satisfaction. For this study, the authors preferred higher scores to indicate higher satisfaction, as this format is also considered in the literature [12].

## Results

**Fig. 2:**
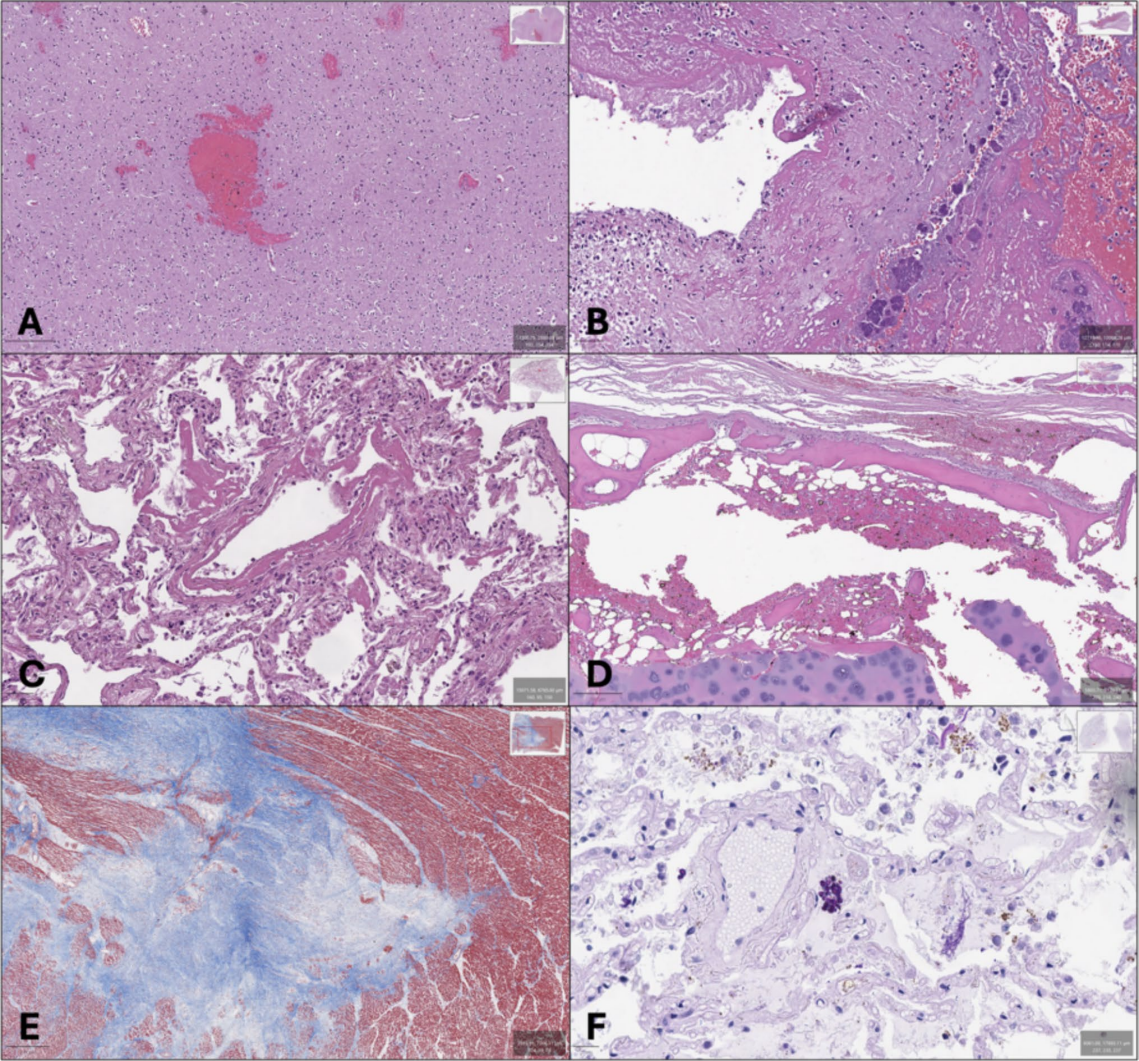
A: WSI 1 – Brain H&E; Intraparenchymal hemorrhage. B: WSI 39 – Aortic valve H&E: Bacterial endocarditis. C: WSI 33 – Lung H&E; Hyaline membranes. D: WSI 52 – Larynx H&E; Cartilage and muscular hemorrhage. E: WSI 91 – Heart Masson; Myocardial fibrosis. F: WSI 81 – Lung PAS; Fungal spores

One hundred glass slides of forensic interest, including different organs and tissues, have been selected. The manner of death included homicide (10 slides), suicide (15), natural (30 slides), accidental (15 slides), and medical liability (30 slides). Eighty slides were stained with standard hematoxylin and eosin, and 20 with histochemical stains (PTAH, ALCIAN-PAS, PAS, Masson Trichrome, Perls, Von Kossa) and immunohistochemical markers (CD20, CD31, MPX, CD61, CD163, CD34, CD3, CD5, CD68, CD15, alpha-smooth muscle actin). Table 1 shows tissues, stains, and synthetic histopathological diagnoses. Figure 2 displays some of the WSI analyzed.

**Table 1:** List of glasses examined by pathologists. N: case number, H&E: hematoxylin–eosin; αSMA: alpha-smooth muscle actin; + : weak expression; +  + : moderate expression; +  +  + : strong expression; -: no expression

N	Tissue	Stain	Diagnosis
1	Brain	H&E	Subarachnoid and intraparenchymal hemorrhage
2	Liver	H&E	Steatosis and lymphocytic infiltration
3	Lung	H&E	Alveolar hemorrhage
4	Brain	H&E	Intraparenchymal hemorrhage
5	Lung	H&E	Edema
6	Heart	H&E	Myocardial fibrosis
7	Heart	H&E	Wavy fibers
8	Brain	H&E	Edema and vascular stasis
9	Thrombus	H&E	Ante-mortem thrombus
10	Lung	H&E	Pulmonary thrombo-embolism
11	Lung	H&E	Subpleural hemorrhages
12	Heart	H&E	Adipositas cordis
13	Spleen	H&E	White pulp hyperplasia
14	Liver	H&E	Macrovascular steatosis
15	Heart	H&E	Subepicardial hemorrhages
16	Lung	H&E	Subpleural hemorrhages
17	Uterus	H&E	Endometrial crumbling
18	Skin	H&E	Subcutaneous hemorrhage
19	Bone	H&E	Absence of pathology
20	Coronary artery	H&E	Atherosclerosis
21	Heart	H&E	Perivascular lymphocytic infiltrates
22	Ovary	H&E	Luteal phase
23	Lung	H&E	Pulmonary edema
24	Adrenal gland	H&E	Medullary hemorrhage
25	Lung	H&E	Hyaline membranes
26	Lung	H&E	Alveolar hemorrhage
27	Coronary artery	H&E	Critical stenosis
28	Thyroid	H&E	Follicular hemorrhages
29	Thyroid	H&E	Absence of pathology
30	Coronary artery	H&E	Calcified concentric stenosis
31	Aorta	H&E	Dissection
32	Coronary artery	H&E	Eccentric stenosis
33	Lung	H&E	Hyaline membranes, alveolar hemorrhages
34	Thyroid	H&E	Perifollicular hemorrhages
35	Kidney	H&E	Sub-capsular hemorrhage
36	Lung	H&E	Intrabronchial foreign bodies
37	Heart	H&E	Lymphocytic myocarditis
38	Kidney	H&E	Glomerulonephritis
39	Aortic valve	H&E	Bacterial endocarditis
40	Coronary	H&E	Critical thrombosed stenosis
41	Heart	H&E	Cytoplasmatic vacuolization
42	Cerebellum	H&E	Subarachnoid hemorrhage
43	Skin	H&E	Subcutaneous hemorrhage
44	Muscle	H&E	Muscular hemorrhages
45	Supra-aortic trunks	H&E	Dissection
46	Aorta	H&E	Intramural hematoma
47	Lung	H&E	Fatty and bone marrow embolism
48	Pulmonary vessels	H&E	Pulmonary thromboembolism
49	Brainstem	H&E	Subarachnoid and intraparenchymal hemorrhage
50	Thyroid	H&E	Interstitial hemorrhage
51	Skin	H&E	Dermal hemorrhage
52	Larynx	H&E	Cartilage and muscular hemorrhage
53	Coronary artery	H&E	Eccentric plaques
54	Lung	H&E	Pneumonia
55	Thyroid	H&E	Subcapsular hemorrhages
56	Lung	H&E	Interstitial pneumonia
57	Skin	H&E	Electric mark
58	Heart	H&E	Myocardial fibrosis
59	Spleen	H&E	Red pulp hyperplasia
60	Kidney	H&E	Inflammatory caverns
61	Kidney	H&E	Vascular stasis
62	Adrenal gland	H&E	Absence of pathology
63	Lymph node	H&E	Central necrosis
64	Kidney	H&E	Post-mortem ischemic changes
65	Bowel	H&E	Wall perforation without inflammatory infiltrate
66	Bowel	H&E	Perilesional hemorrhage
67	Lung	H&E	Small vessels vasculitis
68	Lung	H&E	Lung adenocarcinoma and perilesional hemorrhage
69	Esophagus	H&E	Submucosal hemorrhage
70	Ileus	H&E	Mucosal hemorrhage
71	Bone marrow	H&E	Absence of pathology
72	Pericardium	H&E	Lymphocytic infiltration
73	Stomach	H&E	Absence of pathology
74	Lung	H&E	Pneumonia
75	Thyroid	H&E	Absence of pathology
76	Lung	H&E	Interstitial pneumonia and acute lung injury
77	Esophagus	H&E	Absence of pathology
78	Aorta	H&E	Dissection
79	Ovary	H&E	Absence of pathology
80	Lung	H&E	Hemorrhagic pneumonia
81	Lung	PAS	+ + +
82	Lung	ALCIAN-PAS	+ +
83	Lung	CD20	+ + + vessels
84	Lung	CD31	+ + + vessels
85	Bone marrow	MPX	+ + +
86	Lung	Von Kossa	+ + + interstitial
87	Lung	CD163	+ + + alveolar
88	Bone marrow	CD34	-
89	Lung	CD61	+ + lung vessels
90	Heart	CD3	+ + epicardium
91	Heart	Masson Trichrome	Fibrosis
92	Lung	CD5	+ perivascular
93	Lung	CD68	+ + + perivascular
94	Lung	CD15	+ + perivascular
95	Lung	PTAH	+ +
96	Aorta	αSMA	+ + +
97	Lung	PAS	+ +
98	Lung	Perls	+ +
99	Bone	Masson Trichrome	No pathology
100	Aorta	Masson Trichrome	Cystic degeneration

Scan times averaged 44 s per slide, and storage sizes ranged from 119 to 997 Megabytes (with a mean of 640 Megabytes per slide). All pathologists reported high-performance scores for the system’s reliance on glass slides, image quality (including resolution and color fidelity), and ease and efficiency of digital slide navigation (supplementary material contains downloadable WSI 57—H&E—Skin with electric mark).

Side-by-side comparisons between diagnoses performed on the microscope versus WSIs on a widescreen were excellent, with a mean concordance of 97.8% (Table 2).

**Table 2:** Intra-operator concordance between diagnoses performed on the microscope versus WSIs on a widescreen (mean concordance: 97.8%) and CSUQ V.3 questionnaire results (mean score: 6.6/7). Diagnosis concordance between glass and WSIs was marked when each pathologist reported an identical diagnosis in both examinations

	Pathologist 1	Pathologist 2	Pathologist 3	Pathologist 4	Pathologist 5	Pathologist 6
Concordance glass/WSI diagnosis	98/100 98%	95/100 95%	99/100 99%	98/100 98%	100/100 100%	97/100 97%
CSUQ Version 3 questionnaire	6.7/7	6.4/7	6.8/7	6.6/7	6.9/7	6.4/7

CSUQ Version 3 questionnaire showed high satisfaction for all pathologists (mean score: 6.6/7).

## Discussion

Digitalization is transforming nearly every aspect of medicine, ushering in a new era marked by greater efficiency, precision, and accessibility in healthcare. From electronic health records and telemedicine to artificial intelligence and data analytics, digital tools are changing how healthcare professionals work [13].

In this context, the introduction of digital technology for acquiring stained tissue sections on glass slides has profoundly impacted the pathology field. Nowadays, increasingly more pathology diagnosis and research services worldwide consider WSIs the mainstream option in daily caseload management [14].

WSIs, often called digital or virtual pathology, is a technique that captures high-resolution digital images of entire stained tissue sections from glass slides using high-technology dedicated scanners. These images can then be viewed on a computer with a dedicated monitor for reporting, characterized by high resolution, tone balancing systems, and a high refresh rate, enabling pathologists to examine WSIs with magnification and spatial navigation similar to traditional microscopy [10]. This technique, combined with other digital tools such as barcoding, specimen tracking, and digital dictation, can significantly improve a pathology department’s safety, quality, and efficiency [15].

Considering the unique potential of this technique, the scarcity of literature on WSIs in forensic pathology, and the lack of formal validation in this specialty, our study represents the first multicenter effort to evaluate the application of WSIs in the forensic pathologist’s workflow.

Our findings revealed consistently high-performance ratings from all participating pathologists, notably in terms of glass slide reliability, digital image quality, and the efficiency and ease of digital slide navigation. These results highlight a strong endorsement from practitioners for integrating this technology into routine forensic pathology practice.

Further, the CSUQ Version 3 indicated a high level of satisfaction among all pathologists, underscoring excellent usability perceptions.

Finally, side-by-side comparisons of diagnoses performed on the microscope versus WSIs on a widescreen showed excellent concordance, aligning with the diagnostic accuracy standards established by the College of American Pathologists [10, 11].

Overall, our findings demonstrate the positive reception of WSIs among practitioners and their non-inferiority to traditional microscopy for diagnostic accuracy. Thus, this validation study supports the adoption of WSIs in forensic pathology, with promising implications for advancing service efficiency, research, and educational opportunities in the field.

In service delivery, digital forensic histopathology brings transformative efficiencies to forensic workflows. High-quality digital slides can provide detailed images that can be quickly shared with specialists in different locations, breaking down geographical barriers and enabling real-time consultations. This instant sharing capability is invaluable in forensic pathology, where time-sensitive cases such as legal inquiries, public health risks, or criminal investigations require fast and accurate conclusions. Pathologists can instantly share digital images of tissue samples with experts in specialized fields, such as neuropathology, allowing for a more comprehensive assessment of complex cases. This collaboration may accelerate the diagnostic process and improve the accuracy of conclusions drawn, as multiple experts can provide insights without the delays that traditionally accompany physical sample transfers [16]. Digital forensic histopathology can support a more robust, accurate, and efficient diagnostic process. Integrating digital sharing, automated analysis, and detailed documentation into routine practice may enable forensic pathology services to provide faster and more reliable results, an essential feature in the context of legal and public health inquiries where accuracy and efficiency are paramount.

In the research field, digital forensic histopathology offers a way to study specific features of forensic cases, especially in large-scale data analysis and retrospective studies. This capability is particularly beneficial in forensic science, where reanalyzing older cases may provide new insights.

In addition, advanced algorithms and machine learning models, being at the heart of current digital pathology research, may allow researchers to analyze forensic histopathological patterns in ways traditional microscopy cannot. For example, artificial intelligence (AI) models can be trained to detect tissue abnormalities or specific markers of disease or injury. These models can also quantify cellular density, tissue architecture, or inflammatory responses. This quantitative analysis reduces human subjectivity and introduces statistical rigor into forensic histopathology, making findings more reproducible and reliable [17].

Moreover, such findings can have broader implications, influencing public health policies or legal standards. Thus, the ability to analyze vast amounts of data with digital tools may accelerate forensic research, opening new avenues for discovery and improving the scientific rigor underpinning forensic pathology practices.

Finally, digital forensic histopathology may reshape forensic pathology education by harnessing the power of digital imaging and interactive learning tools. The first advantage is the standardization of digital images, which avoids discrepancies in student, resident, and fellow testing and scores.

In addition, WSIs can allow students and trainees to access high-resolution images without being physically present in a laboratory. Indeed, traditional histopathology teaching relies heavily on in-person observation through microscopes, which limits accessibility and can be challenging for students who cannot physically be on-site. With digital platforms, students can remotely access these materials, enabling flexible learning and reaching a more diverse range of learners. These educational innovations also support collaborative learning. Digital platforms may allow students to discuss findings in real-time or asynchronously, often in global, cross-institutional settings. Students can work together on complex cases, consult with experts remotely, and receive feedback on their diagnostic approaches. In this sense, digital forensic histopathology may contribute to a more dynamic, connected learning environment [18, 19].

## Conclusion

Our multicenter study represents the first successful validation of WSIs in forensic pathology. Results were highly satisfactory in terms of slide reliability, digital image quality, navigational efficiency, and user interaction with the device. Additionally, diagnostic concordance between tissue glass slides on the microscope and widescreen digital viewing was excellent.

The anticipated broader adoption of WSIs in forensic pathology holds significant promise for advancing service quality, research capabilities, and educational opportunities. By enhancing diagnostic accuracy and process efficiency, WSIs align well with the precision and reliability essential to forensic pathology investigations.

## Data Availability

The data analyzed during the current study are available from the corresponding author upon reasonable request.
